# Cholesterol metabolism: physiological regulation and diseases

**DOI:** 10.1002/mco2.476

**Published:** 2024-02-24

**Authors:** Jiarui Guo, Silong Chen, Ying Zhang, Jinxia Liu, Luyang Jiang, Lidan Hu, Ke Yao, Yibo Yu, Xiangjun Chen

**Affiliations:** ^1^ Eye Center of the Second Affiliated Hospital Zhejiang University School of Medicine Hangzhou Zhejiang Province China; ^2^ Institute of Translational Medicine Zhejiang University School of Medicine Hangzhou Zhejiang Province China; ^3^ National Clinical Research Center for Child Health The Children's Hospital Zhejiang University School of Medicine Hangzhou Zhejiang Province China

**Keywords:** cholesterol, cholesterol metabolism, diseases, efflux, reverse cholesterol transport, regulation

## Abstract

Cholesterol homeostasis is crucial for cellular and systemic function. The disorder of cholesterol metabolism not only accelerates the onset of cardiovascular disease (CVD) but is also the fundamental cause of other ailments. The regulation of cholesterol metabolism in the human is an extremely complex process. Due to the dynamic balance between cholesterol synthesis, intake, efflux and storage, cholesterol metabolism generally remains secure. Disruption of any of these links is likely to have adverse effects on the body. At present, increasing evidence suggests that abnormal cholesterol metabolism is closely related to various systemic diseases. However, the exact mechanism by which cholesterol metabolism contributes to disease pathogenesis remains unclear, and there are still unknown factors. In this review, we outline the metabolic process of cholesterol in the human body, especially reverse cholesterol transport (RCT). Then, we discuss separately the impact of abnormal cholesterol metabolism on common diseases and potential therapeutic targets for each disease, including CVD, tumors, neurological diseases, and immune system diseases. At the end of this review, we focus on the effect of cholesterol metabolism on eye diseases. In short, we hope to provide more new ideas for the pathogenesis and treatment of diseases from the perspective of cholesterol.

## Graphical abstract

At present, increasing evidence suggests that abnormal cholesterol metabolism is closely related to various systemic diseases, including cardiovascular disease, tumors, neurological conditions immune system disorders, and eye diseases. Cholesterol overload caused by abnormal cholesterol metabolism can induce elevated oxidative stress, heightened inflammatory responses, reduced autophagy, and increased apoptosis in cells through various signaling pathways, ultimately accelerating the development of diseases. Therefore, this review will summarize the relationship between cholesterol metabolism and common diseases and aim to provide new perspectives for the pathogenesis of diseases.

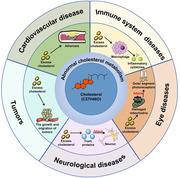

## 1 INTRODUCTION

Cholesterol (C_27_H_46_O) was separated from human gallstones over two centuries ago, capturing the attention of scientists and clinicians. Its physiological and pathological significance is undeniable. Like other sterols, cholesterol is predominantly hydrophobic and biosynthesized in mammalian cells. Mainly found on the cell membrane, cholesterol interacts with adjacent lipids, regulating the rigidity, fluidity, and permeability of the bilayer. Additionally, cholesterol can bind to various transmembrane proteins, aiding in maintaining or altering their conformation. Apart from its role in the membrane structure and function, cholesterol generates diverse oxysterols through both enzymatic and nonenzymatic pathways, with some subsequently metabolized into bile acids.^1^ Oxidative cleavage of cholesterol side chains produces pregnenolone, a common precursor for all other steroid hormones. These derivatives of cholesterol actively participate in various biological processes. Considering the vital role of cholesterol, disruptions in its metabolism may lead to various congenital human diseases.

Recently, a series of discoveries has contributed to a new understanding of cholesterol metabolism. Cholesterol metabolism in the body comprises four parts: biosynthesis, uptake, efflux, and storage. Disruption in any part of the metabolic process is highly likely to lead to the occurrence of acquired diseases. Currently, significant progress has been made in cardiovascular disease (CVD) research. Researchers have discovered various protective mechanisms to reduce atherosclerosis (AS) and attempted to develop drugs targeting cholesterol metabolism.^2^ Nevertheless, the impact of abnormal cholesterol metabolism in the development of other diseases remains unclear. There is increasing evidence suggesting a close relationship between cholesterol metabolism and certain diseases.^3^, ^4^, ^5^, ^6^ Especially in eye diseases, for example, studies have shown that genetic variations in lipid metabolism and transport genes—such as liver lipase, cholesterol ester (CE) transferase, apolipoprotein (Apo) E and ATP binding cassette (ABC) transport protein A1—are associated with an increased risk of age‐related macular degeneration (AMD)^7^, ^8^, ^9^; and the therapeutic effect of reverse cholesterol transport (RCT) pathway in eye diseases also has become a recent research hotspot^10^; in addition, the function of cholesterol in the intraocular lens is different from that in other tissues and organs. These make eye disease the focus of this review. Therefore, in this review, we discuss separately the relationship between cholesterol metabolism and common diseases and aim to provide new perspectives for the pathogenesis of diseases.

In this review, we initially describe the metabolic process of cholesterol in the human body, including synthesis, transport, and efflux. Subsequently, we examine separately the impact of abnormal cholesterol metabolism on common diseases and potential therapeutic targets for each disease, including CVD, tumors, neurological conditions, immune system disorders, and eye diseases.

## 2 CHOLESTEROL METABOLISM IN THE BODY

In this review, we will describe the primary pathways and regulations of various key aspects of cholesterol metabolism, establishing a crucial foundation for understanding the relationship between cholesterol metabolism and diseases in the subsequent text. For more intricate regulatory mechanisms of cholesterol, please refer to the relevant literature on cholesterol regulation.

### 2.1 Biosynthesis and regulation

Cholesterol is a common lipid molecule essential for normal bodily function.^11^ It originates from two pathways: in situ biosynthesis and systemic circulation. For example, in the retina, approximately 28% of cholesterol comes from systemic circulation due to the separation of the blood–retinal barrier, while the remaining cholesterol is synthesized in situ.^12^ Here, we discuss the in situ biosynthesis of cholesterol in detail. Cholesterol biosynthesis begins with acetyl‐CoA, which is converted into mevalonate by 3‐hydroxy‐3‐methylglutaryl coenzyme reductase (HMGCR), a rate‐limiting enzyme in cholesterol biosynthesis. Mevalonate is then converted into squalene, subsequently transformed into lanosterol. Cholesterol biosynthesis is then divided into two pathways: the Bloch and Kandutsch–Russell pathways. In the Bloch pathway, lanosterol undergoes five steps to generate desmosterol, and in the Kandutsch–Russell pathway, lanosterol undergoes five steps to generate 7‐dehydrodesmosterol. Finally, 24‐dehydroxysterol reductase (DHCR24) and 7‐dehydroxysterol reductase (DHCR7) catalyze the last step of both pathways to produce cholesterol.^13^, ^14^

**FIGURE 1:**
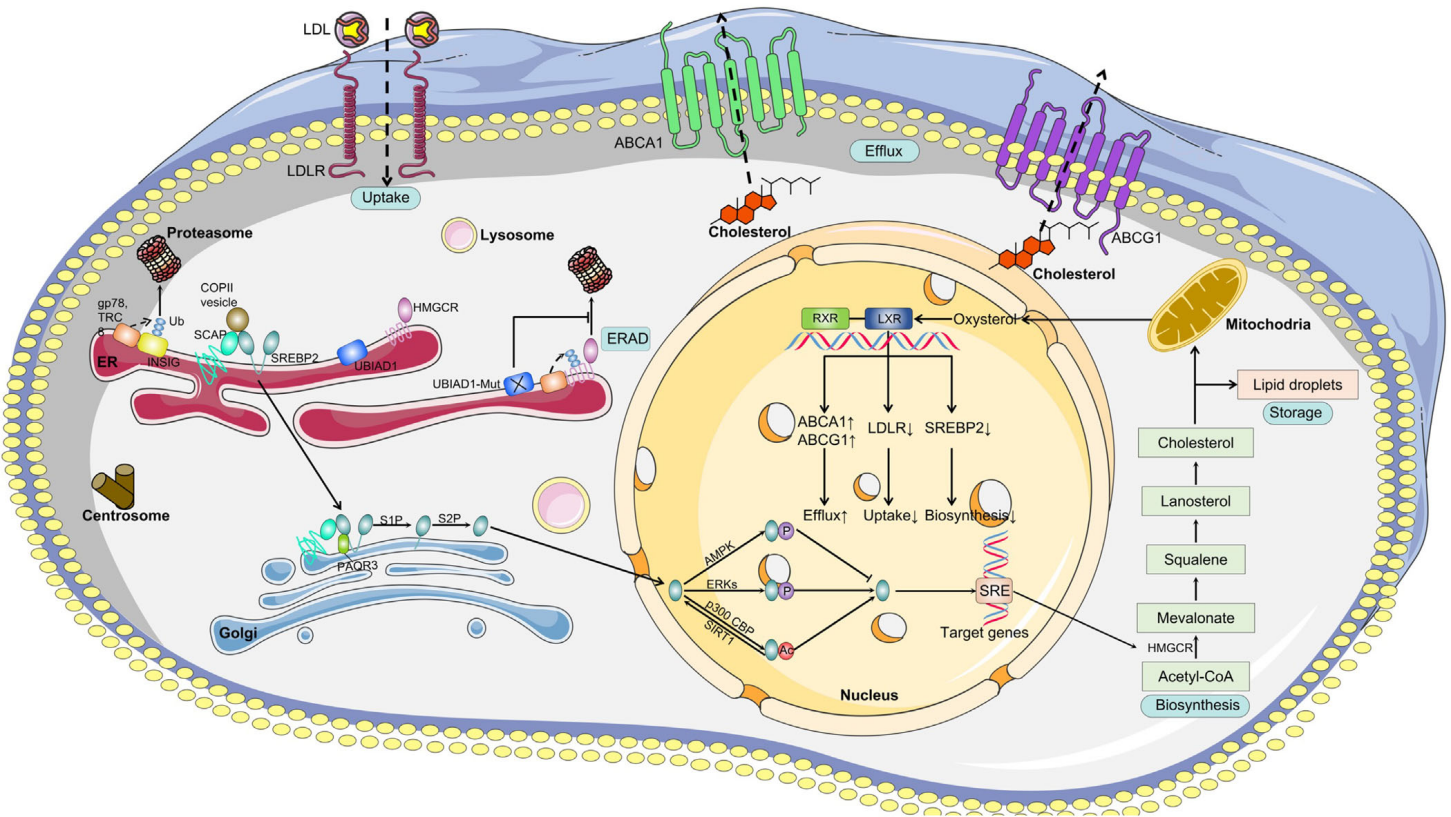
The regulation of cholesterol metabolism in the body. When cholesterol concentration is low, INSIGs are ubiquitylated by gp78 and TRC8, then SCAP interacts with SREBP2 and COPII, allowing the SCAP‐SREBP complex to anchor to the Golgi apparatus via PAQR3, where SREBP2 undergoes proteolytic cleavage of S1P and S2P, entering the nucleus. In the nucleus, SREBP2 interacts with the SRE sequence of promoters of target genes, such as HMGCR, which promotes cholesterol biosynthesis. The generated cholesterol is stored in the form of lipid droplets, and excess cholesterol is excreted out of the cell through the RCT pathway. In the RCT pathway, cholesterol is transported to the mitochondria and activates LXRs. Under normal physiological conditions, the transcription activity of LXRs increases in response to the increase of cholesterol cell level, which decreases the level of cholesterol via multiple pathways. Eventually, excess cholesterol is excreted through the ABCA1 and ABCG. When the concentration is high, SCAP interacts with INSIGs, leading to the blockage of the translocation. HMGCR can also be ubiquitinated and degraded in proteasomes through the ERAD. In addition, when UBIAD1 mutates, it can delay the degradation of HMGCR. COPII, coatomer II; PAQR3, adipoQ receptor 3; S1P, site 1 protease; S2P, site 2 protease; SRE, sterol regulatory element; Ac, acetyl group; P, phosphate group; Ub, ubiquitin; UBIAD1, UbiA prenyltransferase domain‐containing protein‐1; ERAD, ER‐associated degradation; RXR, retinoid X receptor; LXR, liver X receptor; LDL, low‐density lipoprotein; LDLR, low‐density lipoprotein receptor.

However, cholesterol biosynthesis is a high energy‐consuming process that requires strict regulations. In this section, we briefly review the crucial role of sterol regulatory element‐binding proteins (SREBPs), especially SREBP2, encoded by *Srebp2*, which enhances the expression of genes related to cholesterol biosynthesis in cholesterol biosynthesis, such as HMGCR.^15^, ^16^ SREBP2 is initially produced as an endoplasmic reticulum (ER)‐anchored precursor and becomes activated only after translocating from the ER to the Golgi apparatus.^17^ However, the activity of SREBP2 is regulated by the cellular cholesterol concentration. When cholesterol concentration is high, SREBP cleavage‐activating protein (SCAP) binds to SREBP2 precursors in the ER membrane and interacts with insulin‐induced gene proteins (INSIGs), leading to the SCAP‐SREBP complex being trapped in the membrane and the blockage of the translocation.^18^, ^19^ When the concentration is low, INSIGs are degraded by the ubiquitin–proteasome pathway. SREBP2 precursors then move from the ER to the Golgi, releasing the amino‐terminal active domain through the cleavage.^20^ In this process, nuclear SREBP undergoes post‐translational modifications. The acetylation of P300 and CBP can enhance the expression of nuclear SREBP,^21^ whereas deacetylation of Sirtuin1 (SIRT1) and phosphorylation of AMP‐activated protein kinase (AMPK) can inhibit its expression.^22^, ^23^ These proteins regulate the transcription of SREBP2 (Figure 1). In addition, mTOR complex 1 (mTORC1) also plays an important role in the regulation of SREBP2. When mTORC1 is inactivated, cell autophagy and endosomal membrane trafficking increase, leading to the rise in cholesterol levels in the ER, which will inhibit SREBP2. By contrast, it reduces cholesterol levels in the ER and activate SRBEP2.^24^ A recent study reported that mTORC1 can prevent Lipin1 from entering the nucleus by phosphorylating Lipin1 and promote the expression of SREBP2.^25^

### 2.2 Conversion and transport

As widely known, blood lipids in humans, including cholesterol and triglycerides, are insoluble. Therefore, cholesterol transport in the blood requires association with lipoproteins. In systemic circulation, lipoproteins are classified into five types based on their densities: chylomicrons (CM), very low‐density lipoprotein (VLDL), intermediate‐density lipoprotein (iIDL), low‐density lipoprotein (LDL), and high‐density lipoprotein (HDL), which can convert into each other. Cholesterol in humans comes not only from endogenous biosynthesis but also from external intake. The epithelial cells of the intestine can absorb cholesterol from dietary sources through the Niemann‐Pick type C1‐like 1 protein (NPC1L1), releasing exogenous cholesterol in the form of CM.^26^ Subsequently, CM can be transported to the liver through blood circulation and converted into VLDL, the main function of which is to transport endogenous cholesterol. Ultimately, VLDL is transformed into LDL in the blood, transporting cholesterol throughout the body, including the brain and eyes. In this process, low‐density lipoprotein receptor (LDLR), a cell surface glycoprotein, plays a crucial role in peripheral cells' uptake of cholesterol from systemic circulation, and its transcription can be also activated by SREBP2. Numerous studies have shown that VLDL and LDL are risk factors for large‐vessel diseases, such as AS. And they are positively correlated with the risk of diseases. However, HDL is commonly regarded as “good cholesterol,” because it transports excess cholesterol to the liver for metabolism and elimination.^27^

The protein components of lipoproteins include ApoA, ApoB, ApoC, ApoD, and ApoE. ApoA1 is the major component of HDL, which exerts a prospective effect by transporting cholesterol from peripheral cells to sites of catabolism and activating lecithin‐cholesterol acyl transferase (LCAT) in the RCT pathway. Studies have shown that ApoA1 can boost cholesterol efflux via the ABCA1.^28^ Furthermore, ApoA1 can be upregulated by liver X receptors (LXRs) in a dose‐dependent manner.^29^ ApoB is the major component of CM, VLDL, and LDL, which are involved in the lipid delivery. Some data strongly suggest that the risk of CVD is positively correlated with the concentration of ApoB. ApoB has two subtypes, ApoB48 and ApoB100, produced in the intestine and liver respectively. ApoB100 plays an important role in inducing LDL formation and promoting inflammation.^30^ And ApoE is responsible for cholesterol transport between cells or tissues,^31^ directly regulated by LXRs in macrophages.^32^ In blood, ApoE mainly combines with CM and VLDL; however, in the brain and retina, ApoE‐containing lipoproteins have densities similar to those of HDL.^31^ It has three isoforms (2, 3, and 4) in the human body. ApoE4 increases the risk of Alzheimer's disease (AD) and reduces the risk of AMD, whereas ApoE2 has the opposite effect, which is secreted by the retinal pigment epithelium (RPE) and increases significantly after exposure to complement in the drusen.^33^, ^34^, ^35^, ^36^ This is because ApoE2 has obvious defects in cholesterol transport compared with ApoE4.^37^ In addition, ApoE4 can maintain cholesterol homeostasis by regulating cholesterol efflux, preventing the autophagy deficiency and mitochondrial damage caused by complement activation.^37^

### 2.3 Efflux

Almost all cells in the human body produce cholesterol; however, only a few cells can metabolize it. Therefore, to maintain cholesterol homeostasis in cells, excess cholesterol must be expelled, transported to the liver through the systemic circulation, metabolized, and ultimately discharged. This process is also known as the RCT. Cholesterol efflux from cells is an early step of the RCT, in which LXRs and ABCA1/ABCG1 as key regulators play a crucial role (Figure 1).

LXR, a ligand‐activated nuclear transcription factor, has the opposite effect to SREBP2, activated when cells accumulate excess cholesterol. The two highly homologous LXR proteins in the human body are distributed in different tissues. LXRα is primarily found in the intestine, liver, kidney, fat, and macrophages, while LXRβ is widely distributed in almost all cells.^38^, ^39^, ^40^ In the nucleus, LXR associates with retinoid X receptor (RXR) to form the obligate heterodimer. Upon binding LXR agonists, the heterodimer activates the transcription of target genes, such as ABCA1, ABCG1, SREBP‐1c, and ApoE.^41^ In addition, LXR has other functions in the human body. For example, in animal models of diabetes, LXR agonists regulate the metabolism of glucose and reduce blood glucose levels by upregulating the glucose transporter 4 (GLUT4).^42^ Furthermore, LXR is one of the key receptors regulating the inflammatory response,^43^ which can inhibit the expression of inflammatory factors (such as NF‐κB).^44^ LXR also regulates cell apoptosis and autophagy, which will be discussed later. We then discuss the key downstream targets of LXR: ABCA1 and ABCG1. ABCA1 and ABCG1 utilize ATP as an energy source to regulate the transmembrane transport of lipids, sugars and amino acids. ABCA1 is a complete transporter protein widely expressed throughout the body. It consists of two tandem repeats of transmembrane domains, each with six transmembrane segments and a large glycosylated extracellular domain. In the RCT, the main role of ABCA1 is to transfer intracellular cholesterol to lipid‐free ApoA1 when activated by LXR,^45^ resulting in the formation of nascent HDL. Subsequently, LCAT induces the formation of mature HDL.^28^ Studies have shown that the inactivation or mutation of ABCA1 can significantly reduce the efflux of cellular cholesterol to ApoA1. Therefore, patients with Tangier disease or *Abca1*−/− mice cannot form mature HDL, and their plasma total cholesterol (TC) is very low, indicating abnormal lipid deposition in various tissues.^46^ ABCG1, as a half‐sized transporter, is widely expressed in macrophages and other cell types, except hepatocytes and enterocytes.^47^ Studies have shown that mice with *Abcg1* deletion show cholesterol accumulation in macrophages and the liver.^47^ In contrast, the tissues of ABCG1 overexpression mice were protected from lipid accumulation induced by a high‐fat diet.^48^

Cholesterol metabolism is an intricate process that not only depends on the synchronized coordination of various steps but is also regulated by multiple signaling pathways throughout the body. The various diseases we will discuss later are largely a result of disordered cholesterol metabolism, leading to the loss of compensatory mechanisms and thus accelerating disease progression.

## 3 CHOLESTEROL METABOLISM IN CVDs

Over the past few decades, substantial strides have been achieved in treating CVD, marked by heightened public health awareness, a stronger focus on disease prevention and advancements in percutaneous coronary intervention and stent development. However, CVD persists as the leading global cause of death,^49^ driving ongoing research into its pathogenesis. AS is consistently recognized as the linchpin of CVD, serving as the pathophysiological mechanism underlying ischemic heart disease, stroke, peripheral artery disease, and aneurysm formation. Presently, clinical treatment primarily centers on mitigating complications associated with AS using drugs that lower LDL levels, such as statins.^50^ Nevertheless, the impact of this multifaceted disease on global mortality underscores the necessity for novel targeted therapies.

To discover new targeted drugs, understanding the pathogenesis of AS is crucial. The formation of foam cells, resulting from the accumulation of atherosclerotic lipoproteins induced by monocyte‐derived macrophages, is a pivotal early sign of AS. In simpler terms, macrophages play a key role in the pathogenesis and development of AS.^51^ Normally, cholesterol metabolism in macrophages remains stable. Disruption of metabolism can activate various signaling pathways associated with inflammation and cell apoptosis. Each pathway regulates multiple downstream genes related to inflammation, oxidative stress and cell cycle. This significantly promotes the maintenance and progression of AS.^52^ Therefore, we can conclude that the central focus of AS treatment will be reducing cholesterol accumulation.

Because the RCT pathway is presently regarded as the sole mechanism for clearing excess cholesterol in the human body, there has been a growing focus on the RCT research in recent years. Researchers have identified numerous drugs that enhance the pathway,^53^, ^54^ but the most noteworthy development is the emergence of microRNAs (miRNAs). miRNAs are endogenous noncoding RNA molecules, typically 21–25 nucleotides in length. They generally bind to the 3′ untranslated regions of target genes to repress mRNA translation, regulating various biological processes such as cell differentiation, proliferation, metabolism, and death.^55^ According to reports, miRNAs play a role in maintaining cholesterol homeostasis. For example, miR‐33, ‐144, and ‐148 can regulate the RCT pathway by reducing ABCA1 expression.^56^ Recently, miRNAs have been regarded as the new target for treating AS due to their important regulatory roles. First, miRNAs can decrease the expression of ABCA1 and inhibit the RCT pathway. Research has shown that *ApoE* deficient mice treated with miR‐20a/b exhibits reduced ABCA1 expression in the liver and decreased the RCT in vivo. Additionally, miR‐20a/b inhibits HDL formation and promotes AS development. Conversely, miR‐20a/b inhibitors can increase ABCA1 expression and cholesterol efflux, reduce cholesterol content, and inhibit foam cell formation.^57^ Similar effects have been observed with other miRNAs, such as miR‐302a, miR‐320b, and miR‐613.^58^, ^59^, ^60^ Second, inhibiting or knocking out miRNAs can alleviate inflammation in macrophages treated with oxidized low‐density lipoproteins (ox‐LDL). Studies have found that inhibiting miR‐652‐3p can reduce foam cell formation and decrease IL‐1β, IL‐6, and TNF‐α in macrophages treated with ox‐LDL, which have proven that inhibiting miR‐652‐3p can regulate lipid metabolism and inflammatory cytokine secretion in macrophages, ultimately alleviating AS.^61^ Third, inhibiting or knocking out miRNAs can inhibit oxidative stress in macrophages. For instance, knocking down MiR‐34a‐5p can inhibit cholesterol accumulation and oxidative stress damage.^62^ These results may contribute to the development of new therapies related to the RCT‐based treatment strategies. However, the specific mechanism of miRNA is still unclear, and it is uncertain whether their effects on inflammation and oxidative stress are related to cholesterol efflux. In the future, we should also strive to discover other pathways that can eliminate cholesterol accumulation and attempt to discover safer and more effective targets.

## 4 CHOLESTEROL METABOLISM IN TUMORS

Cholesterol plays a crucial role in the occurrence and development of tumors due to its ability to drive cell proliferation, migration and invasion. Abnormal regulation of cholesterol metabolism in tumor cells mainly involves upregulation of cholesterol synthesis levels, increased cholesterol uptake and abnormal accumulation of metabolic products. These factors contribute to tumor cell proliferation, survival, invasion, metastasis, and enhanced adaptability to the tumor microenvironment, ultimately promoting the occurrence and development of tumors. Some genes related to cholesterol synthesis show increased activity in tumor tissues, leading to the abnormal activation of cholesterol biosynthesis key enzymes, such as HMGCR and squalene monooxygenase (SQLE).^63^, ^64^, ^65^, ^66^, ^67^, ^68^, ^69^, ^70^, ^71^, ^72^, ^73^ which has a significant impact on the development of tumors (Table 1). Next, we will discuss the impact of cholesterol metabolism on tumors, focusing on its effect in prostate cancer (PC) and hepatocellular carcinoma (HCC).

**TABLE 1:** The effect of overexpression of cholesterol synthases on tumors.

Disease	Enzyme	Signal	Functions	References
HCC	SQLE	ERK signaling↑	The growth and migration of HCC↑	^63^
	SQLE	TGF‐β/SMAD signaling↑	The metastasis and chemoresistance of HCC↑	^64^
PC	HMGCR		The growth of PC↑	^65^
	SQLE		The growth of PC↑	^66^
Gastric cancer	HMGCR	Hedgehog/Gli1 signaling↑	The growth of gastric cancer↑	^67^
Glioblastoma	HMGCR	Hippo signaling↑	The growth and migration of glioblastoma↑	^68^
Breast cancer	HMGCR		The growth and migration of breast cancer↑	^69^
Esophageal squamous cell carcinoma	HMGCR	Ras‐ERK signaling↑	The growth and migration of carcinoma↑	^70^
Lung squamous cell carcinoma	SQLE	ERK signaling↑	The proliferation and metastasis of carcinoma↑	^71^
Bladder cancer	SQLE	P53 signaling↓	The proliferation, migration and invasion of bladder cancer↑	^72^
Colorectal cancer	SQLE	MAPK signaling↑	The proliferation of colorectal cancer↑	^73^

Abbreviations: ERK, extracellular signal‐regulated kinase; HCC, hepatocellular carcinoma; MAPK, mitogen‐activated protein kinase; PC, prostate cancer; SMAD, suppressor of mother against decapentaplegic; TGF‐β, transforming growth factor‐β.

### 4.1 Prostate cancer

PC is the second leading cause of cancer death in men worldwide.^74^ Currently, a large number of studies have clearly shown that hypercholesterolemia may be associated with the risk of developing PC.^75^, ^76^, ^77^ A cholesterol‐lowering diet is a favorable factor against the risk of PC.^78^ In fact, a positive correlation between cholesterol accumulation in prostate tissue and the presence of PC has been discovered for a long time.^79^ Since then, more and more studies have supported the indispensable role of cholesterol in the progression of PC. One hypothesis is that the accumulated cholesterol in PC provides a precursor for androgen synthesis in tumors, which can promote the development of castration‐resistant PC (CRPC).^80^ Therefore, the use of statins or enhancement of ABCA1 expression to lower cholesterol levels can reduce the growth of PC by lowering serum or tumor androgen levels.^81^, ^82^ Meanwhile, studies have shown that cholesterol not only promotes tumor proliferation but also that its metabolite, 27‐hydroxycholesterol (27‐OHC), induces an increase in tumor cell proliferation.^83^ Another hypothesis is that an increase in cholesterol levels supports the growth of PC by providing key membrane components such as lipid rafts.^84^, ^85^ It was also found that epidermal growth factor receptor is a cell membrane binding receptor associated with lipid rafts in PC cells,^86^ its activation can lead to the activation of protein kinase B, which promotes the growth of PC.^87^ A study has found that LXR agonists induce cell apoptosis and inhibit the growth of tumor cells by activating cholesterol efflux to disrupt lipid rafts and reducing the downregulation of AKT signaling in vitro model.^88^

Given that cholesterol accumulation promotes tumor growth, inhibiting the biosynthetic pathway of cholesterol is an attractive direction. In addition to the well‐known statins, such as simvastatin,^89^ alternative methods for cholesterol biosynthesis inhibitors are gradually being explored. 2,3‐Oxidosqualene cyclase (OSC), an enzyme that converts 2,3‐monoepoxysqualene to lanosterol, has become a possible new target. Recently a study found that inhibitor of OSC, RO48‐8071 exerts multiple effects. For example, RO48‐8071 can reduce viability of both hormone‐dependent and CRPC cells and induce apoptosis of cells in vitro.^90^ The role of SQLE is also worth studying. Previous studies have shown that its expression is associated with poor prognosis.^91^, ^92^ Recently, Kalogirou et al.^66^ showed that high SQLE protein expression is associated with adverse outcomes in PC, which contributes to the growth of PC tumors. They also demonstrated that SQLE inhibition effectively reduces in situ tumor growth without affecting the overall cholesterol metabolism of mice.^66^ Moreover, the inhibition of SQLE can effectively suppress lymph node metastasis in PC cells.^93^ These findings emphasize that SQLE may be a potential biomarker and therapeutic target in PC patients. In addition to inhibiting key enzymes in the synthesis pathway, blocking cholesterol esterification has become a strategy for cancer treatment. Previously, it has been found that the inhibitor of acyl‐coenzyme A: cholesterol acyltransferase‐1, avasimibe, can significantly reduce the storage of CEs in lipid droplets and increase intracellular free cholesterol levels, leading to cell apoptosis and inhibiting proliferation.^94^ Later, Lee et al.^95^ further demonstrated that inhibition of cholesterol esterification in mouse models inhibits the development and growth of metastatic PC. The downregulation of the Wnt/β‐catenin pathway after CE depletion was determined, and it was suggested that inhibition of cholesterol esterification could inhibit PC metastasis by impairing Wnt/β‐catenin signaling.^95^ In summary, these studies have offered new opportunities for the treatment of PC.

### 4.2 Hepatocellular carcinoma

HCC is the 6th most common cancer worldwide and the third leading cause of cancer‐related mortality worldwide.^96^, ^97^ HCC is induced by various diseases, including hepatitis B virus infection, alcoholic liver disease, and metabolic syndrome.^98^ Despite progress in the diagnosis and treatment of HCC, the majority of HCC patients still have poor prognosis due to tumor progression or recurrence.^99^ Epidemiological studies have shown that cholesterol intake is an independent risk factor for HCC.^100^ Cholesterol metabolism disorders are often observed in HCC, and the activation of cholesterol synthesis drives tumor development.^101^, ^102^ Therefore, reducing cholesterol levels by inhibiting its biosynthesis or promoting its degradation may be an effective strategy for inhibiting the progression of HCC.

How does the cholesterol overload impact the progression of HCC? Numerous studies currently indicate the role of cholesterol in the development of HCC. First, cholesterol overload in the liver can lead to severe cellular redox imbalance, exacerbating various chronic liver diseases.^103^, ^104^, ^105^ Research results have shown that cholesterol overload can lead to antiapoptotic responses and DNA damage. Additionally, redox imbalance can enhance the carcinogenic effect of typical DNA damage inducers such as N‐nitrosodiethylamine.^106^ Second, there is also a connection between cholesterol and inflammation, as previously mentioned, NF‐κB pathway is a key link in the inflammatory response, playing an important role in promoting HCC by increasing proliferation and preventing cell apoptosis.^107^ Lipopolysaccharides (LPS) are components of Gram‐negative bacterial walls that can activate NF‐κB. In a study aimed at investigating the relationship between inflammation and cholesterol accumulation in HCC cells, the authors found that LPS increases intracellular cholesterol accumulation through the NF‐κB pathway in HCC cells, which in turn promotes LPS/NF‐κB pathway and inflammatory response, indicating the proinflammatory effect of cholesterol in HCC cells.^108^ Finally, a recent study also showed that cholesterol is involved in the autophagy process of HCC cells, and demonstrated that cholesterol inhibits the autophagy degradation of receptor tyrosine kinases via Golgi membrane protein 1 to promoting the metastasis of HCC.^109^ It is evident that cholesterol plays an important role in the development of HCC, and other potential mechanisms are worth further research.

After clarifying their relationship, research on the treatment of HCC has also emerged. As a key enzyme in the cholesterol synthesis pathway, SQLE still plays an important role in the development of HCC. Research has shown that in the later stages of tumors development, the activation of TGF‐β signals promotes tumor occurrence, metastasis, and chemotherapy resistance.^110^ In subtypes of TGF‐β, TGF‐β1 plays a crucial role in fiber formation by activating SMAD2/3 phosphorylation,^111^ while SQLE can activate TGF‐β/SMAD signaling pathway and promote the development of HCC. The study by Zhang et al.^64^ also demonstrated that chemical inhibitors of SQLE significantly inhibit the development of HCC in vitro and in vivo. In addition, LXR is also a potential therapeutic target, which can inhibit tumor growth through multiple pathways. In a study, the authors have demonstrated that LXR agonists can downregulate forkhead box M1 (FOXM1) in HCC cells, thereby inhibiting the expression of cyclin D1 and cyclin B1, leading to cell cycle and proliferation arrest.^112^ Another study also reported that activation of LXR induces the expression of suppressor of cytokine signaling 3, leading to downregulation of cyclin D1 and upregulation of p21 and p27, inhibiting the growth of HCC cells.^113^ However, some studies have also pointed out that chronic activation of LXRα promotes HCC, at least in part, by promoting innate immune suppression as a result of accumulation of oxysterols.^114^ Therefore, caution is needed when exploring LXR activating drugs for HCC treatment. Finally, due to the core role of SREBP2 in regulating cholesterol homeostasis, its inhibition also exhibits antitumor effects.^115^, ^116^

It is evident that an excess of cholesterol in tumors can promote the growth and metastasis of tumor cells. Given the substantial impact of cholesterol and its metabolites on tumors, researching the disruption of cholesterol metabolism in cells within the tumor microenvironment will be crucial in the future. This research will offer substantial assistance in antitumor therapy.

## 5 CHOLESTEROL METABOLISM IN NEUROLOGICAL DISEASES

The brain is the organ with the highest cholesterol content, accounting for approximately 25% of total body cholesterol.^117^ Unesterified cholesterol is the main sterol in the adult brain, along with small amounts of deamination sterols and CEs. Cholesterol is not only a crucial structural component of cell membranes and myelin, but also necessary for synaptic and dendritic formation.^118^, ^119^ The consumption of cholesterol in neurons can impair the exocytosis, neuronal activity and neurotransmission of synaptic vesicles, leading to dendritic spines and synaptic degeneration.^120^, ^121^, ^122^ Therefore, cholesterol is crucial for neuronal physiology, and cholesterol metabolism may be related to some central nervous system (CNS) diseases (Table 2), such as AD and Parkinson's disease (PD).^123^, ^124^, ^125^, ^126^, ^127^, ^128^

**TABLE 2:** The relationship between common neurological disorders and cholesterol metabolism.

Disease	Experimental model	Experimental aim	Conclusion	References
AD	AD mice	The effect of LXR agonist TO901317 on the cognitive function of AD mice.	TO901317 reduces the production of Aβ in the brain by promoting cholesterol efflux.	^123^
	AD mice	The effect of increased cholesterol abundance on neurodegeneration.	The increase in cholesterol content in neurons in the brain may help induce and/or worsen AD.	^124^
		The effect of AD on the ability of ABCA1‐mediated cholesterol efflux.	In AD patients, ability of ABCA1‐mediated cholesterol efflux decreases.	^125^
		The contribution of ApoE4 and astrocytes to amyloidosis in AD.	ApoE4 astrocytes induce amyloidosis through cholesterol oversupply.	^126^
PD	PD mice	The relationship between hypercholesterolemia and dopaminergic neurodegeneration.	Hypercholesterolemia can lead to oxidative stress and mitochondrial dysfunction, inducing dopaminergic neurotoxicity.	^127^
		Levels of cholesterol metabolites in cerebrospinal fluid of PD patients.	7α‐hydroxycholesterol levels are positively correlated with depression in PD patients.	^128^

Abbreviations: Aβ, amyloid proteins‐β; AD, Alzheimer's disease; PD, Parkinson's disease.

### 5.1 Alzheimer's disease

AD is the main cause of dementia in the elderly. The pathological features of AD include the aggregation of Aβ and neurofibrillary tangles, twisted fibers of tau. So far, the amyloid hypothesis has dominated research on AD. However, the repeated failures of clinical trials have challenged our narrow understanding of the pathogenesis of AD, indicating that Aβ may be a prominent pathological feature, but not the only pathogenic factor, and has sparked extensive investigation into the potential mechanisms of AD.^129^, ^130^ An increasing amount of data indicates a close correlation between brain cholesterol deficiency and the pathogenesis of AD, forming a new cholesterol hypothesis.^131^, ^132^ More and more studies also show that AD patients have lower brain cholesterol levels, reduced cholesterol synthesis and trafficking.^133^, ^134^ In addition, due to the presence of the blood–brain barrier, although some studies have shown that people with high plasma cholesterol levels are more prone to AD,^135^, ^136^ we cannot easily conclude that high plasma cholesterol is a risk factor for AD, and further research is needed to elucidate the relationship between cholesterol and AD.

At present, changes in cholesterol metabolism have always been considered a key factor in AD's development.^137^ Most of the cholesterol in the brain is catalyzed by the cytochrome P450 46A1 (CYP46A1) to 27‐OHC and 24‐S‐hydroxycholesterol (24‐OHC). Research has shown that 27‐OHC is a risk factor for the development and exacerbation of AD pathology by disrupting cholesterol metabolism and increasing Aβ in the brain, while 24‐OHC can become a protective factor for AD.^138^ Therefore, the exploration of CYP46A1 has become a hot topic in the treatment of AD. At present, CYP46A1 has been studied in different models. For example, impaired learning and defective hippocampal long‐term potentiation were observed in the hippocampus of *Cyp46a1* knockout mice.^139^ Studies on mice overexpressing CYP46A1 have also found that increased CYP46A1 activity may improve memory.^140^ A recent study showed that activating CYP46A1 improves animal performance in hippocampal dependent MWM and situational fear regulation tests. Improved behavior is accompanied by an increase in 24‐OHC brain content, neuronal cholesterol turnover, and specific presynaptic and postsynaptic proteins. At the same time, an increase in astrocyte response and a decrease in microglial activation were observed.^141^ Therefore, we can believe that increasing the level of 24‐OHC in the brain and the activity of CYP46A1 may be an effective strategy for treating AD. In addition to CYP46A1, statins are also widely used due to their anti‐inflammatory, antioxidant, and antithrombotic properties, which can reduce the risk of AD events and dementia. However, observational studies and randomized controlled trials of AD have shown conflicting results,^142^, ^143^ which also reflect the complexity of the relationship between cholesterol and cognitive decline in the brain. Therefore, further research on the metabolism of cholesterol in the brain may be crucial for understanding how changes in blood cholesterol affect cognitive and neurodegenerative processes.

### 5.2 Parkinson's disease

PD is the second most common neurodegenerative disease globally, characterized by various motor and nonmotor symptoms, including static tremor, rigidity, motor delay, and gait/posture instability.^144^ As widely known, α‐synuclein aggregation and neuroinflammation are the two main pathological features of PD.^145^, ^146^ However, the role of cholesterol in PD has not been appropriately addressed. Some studies have shown that cholesterol plays a role in dopaminergic neurodegeneration, and further evaluation is needed to understand its role in PD.

Although the role of cholesterol in PD is still unknown, epidemiological studies have shown that statins have the lowest risk of PD. They can act as antioxidants and anti‐inflammatory agents to reduce the formation of reactive oxygen species (ROS) and exert neuroprotective effects through various mechanisms, such as inhibiting proinflammatory cytokines, inhibiting microglial cell activation, inhibiting oxidative stress, and reducing α‐syn aggregation.^147^ Although additional randomized controlled trials and observational studies are needed to confirm this conclusion, it also supports the important role of statins. For example, simvastatin can inhibit the activation of NF‐κB pathway, reduce proinflammatory cytokines, restore striatal dopamine (DA) levels, and improve motor function in PD patients.^148^ However, there is still controversy regarding the use of statins. In a prospective study on the relationship between statin use and PD, statin use may be associated with a higher risk of PD, while higher TC may be associated with a lower risk.^149^ These data are inconsistent with the hypothesis that statins can prevent PD. Some studies even suggest that the use of statins may have adverse effects on baseline nigrostriatal DA degradation and long‐term outcomes in PD patients.^150^ In the future, we should enhance our understanding of the pathogenesis of PD and clarify the relationship between the disease and statin therapy.

The above content informs us that the metabolism of cholesterol in the brain is a complex process. There is an urgent need to conduct in‐depth research on the specific role of cholesterol metabolism in neurodegenerative diseases. With the deepening of research, effective intervention measures targeting cholesterol and its metabolites also have important clinical significance for delaying the development of diseases and finding new therapeutic targets.

## 6 CHOLESTEROL METABOLISM IN IMMUNE SYSTEM DISEASES

Diseases of the immune system are mainly caused by the accumulation of a large number of immune cells and immunoglobulins generated by an excessive immune response, which damages normal tissues, causing pathological changes and functional damage. Their occurrence is closely related to the activation of inflammatory reactions. However, abnormal cholesterol metabolism often leads to an enhanced immune response, which seems to suggest that cholesterol also plays an important role in the pathogenesis of immune system diseases. At present, there is currently very little research on cholesterol metabolism and the immune system. Therefore, in this review, we discuss systemic lupus erythematosus (SLE) and rheumatoid arthritis (RA), attempting to explore the role of cholesterol in the development of these diseases.

### 6.1 Systemic lupus erythematosus

SLE is a common and potentially fatal autoimmune disease characterized by multiple organ damage associated with autoantibodies, including the kidneys, cardiovascular system, neurological system and skin, primarily affecting women of childbearing age.^151^ In the past few years, we have made some interesting progress in understanding the pathogenesis of SLE, including metabolic disorders, signaling and biochemical defects in immune cells, as well as impaired perception and repair of DNA damage.^152^ Among these factors, dyslipidemia is a common feature of patients with SLE, and associated with accelerated AS and increased risk of CVD.^153^ Recent studies have shown that the ability of cholesterol efflux is impaired to varying degrees in SLE patients,^154^ which provides new insights into the pathogenesis of SLE. The study found that both ABCA1/ABCG1‐mediated cholesterol efflux is reduced, which may have a significant impact on the formation of foam cells and the inflammatory activation of macrophages.^155^ And they believed that the impairment is related to the dysfunction of HDL. This view has also been verified in another study. They found in vitro that resveratrol can enhance the cholesterol outflow of macrophages through ABCA1 and ABCG1 pathways, effectively resisting the impact of SLE on AS.^156^ Therefore, we may conclude that the occurrence of SLE is also related to the deposition of cholesterol. At the same time, drug treatment for SLE is gradually advancing, including statins.^157^ With the progress of confirmatory trials, its therapeutic effect is worth looking forward to.

### 6.2 Rheumatoid arthritis

RA is also an autoimmune disease in which the immune system mistakenly attacks the host's body systems. Its clinical features typically manifest as chronic noninfectious synovial inflammation, cartilage and bone destruction. At present, there are no effective drugs for RA, and only disease‐relieving antirheumatic drugs and biological agents can be used to alleviate symptoms. However, these drugs can also bring serious side effects during therapy.^158^ Patients with active RA often have the clinical characteristics of the lipid paradox, with lower levels of TC, HDL‐C, and LDL‐C, leading to a higher risk of cardiovascular incidence rate and RA mortality.^159^ The reason for this phenomenon is that, in RA patients, a large amount of cholesterol accumulates in macrophages. Studies have shown that chronic inflammation contributes to the accumulation of lipids in the synovium.^160^ So, how can we change this paradox? Some studies have showed that the suppression of LXRα agonism can improve lipid metabolism and inflammatory process in RA.^161^ Although this contradicts our traditional understanding,^162^ it will also help develop new drugs for treating RA with dyslipidemia.

In summary, given the complexity of immune system diseases, we can only offer a rough overview of the role of cholesterol in their pathogenesis and provide clues for future research. We aim to make more progress in the future to further identify potential therapeutic targets.

## 7 CHOLESTEROL METABOLISM IN EYE DISEASES

At present, there is limited research on the metabolism of cholesterol in the eye. As a common lipid present in the retina, we also have little understanding of its role in retinal function. As both the brain and retina belong to the CNS and share many similarities, numerous studies compare the two. Similar to the brain, due to the blood–retinal barrier, cholesterol in the retina primarily comes from in situ synthesis, and its metabolism is strictly regulated by various mechanisms. In this review, we will discuss not only retinal diseases like AMD and diabetic retinopathy (DR) but also several other common eye conditions, including cataracts, corneal diseases and dry eye syndrome (DES), which are the primary focus of our review (Table 3).^163^ ^—^ ^174^

**TABLE 3:** The relationship between common eye diseases and cholesterol metabolism.

Disease	Experimental model	Experimental aim	Conclusion	References
AMD	The mice of targeted deletion of macrophage *Abca1* and *Abcg1*	The effect of deletion of *Abca1* and *Abcg1* on cholesterol homeostasis.	The lack of *Abca1* and *Abcg1* will lead to cholesterol accumulation and trigger early AMD.	^163^
		The potential function of ABCA1 in the eye.	Impaired activity of the ABCA1‐mediated lipid efflux pathway may contribute to the progression of AMD.	^164^
	Human iPSC‐derived RPE cells of *Abca1* deletion	The identification of conditions for reducing the expression and function of ABCA1 in RPE.	The deletion of *Abca1* leads to intracellular lipid accumulation and promotes the development of AMD.	^165^
	The mice of *Tspo* knockout	The impact of *Tspo* knockout on RPE in mice.	The lack of TSPO in mice leads to the defect of cholesterol efflux in RPE cells and induces the onset of AMD.	^166^
Cataract	Male and female Sprague–Dawley rats	The mechanism of cataract induced by E2012.	The reduction of cholesterol in lens has been proved to be a prerequisite for cataract induced by E2012.	^167^
	Male Sprague–Dawley rats	The relationship between lens opacity and cholesterol biosynthesis inhibition.	The inhibition of cholesterol biosynthesis is one of the reasons for lens turbidity.	^168^
	The knock‐in mouse model with G589S mutation in *Lss*	The roles of LSS in cataractogenesis.	The down‐regulation of cholesterol biosynthesis may lead to the lens development defect during cataract.	^169^
	The Shumiya cataract rat	The mechanism of cataract formation.	Cholesterol in the lens may be important for maintaining normal lens homeostasis.	^170^
Corneal diseases		To understand abnormal cholesterol accumulation in cornea.	LCAT, ApoD, ApoA1 and ABCA1 are essential to prevent cholesterol accumulation and maintain vision.	^171^
DES		The potential correlation between DES and statin or dyslipidemia	History of statin use or dyslipidemia is related to the increased probability of diagnosis of DES.	^172^
	The heterozygous *Ubiad1* G184R knock‐in mice	To study how UBIAD1 promotes the occurrence of SCD.	The *Ubiad1* mutation associated with SCD impairs the transport of ER to Golgi apparatus, finally leads to accumulation of cholesterol in the cornea.	^173^
DR	The mice of ablation of *Cyp46a1*	The role of CYP46A1 in retina and retinal vessels.	The ablation of *Cyp46a1* leads to the increase of cholesterol in the retina and induce some manifestations of early DR.	^174^

Abbreviations: AMD, age‐related macular degeneration; DES, dry eye syndrome; DR, diabetic retinopathy; LSS, lanosterol synthase; SCD, Schnyder corneal dystrophy; TSPO, 18‐kDa translocator protein.

### 7.1 Age‐related macular degeneration

#### 7.1.1 The relationship of AMD and cholesterol metabolism

AMD is the leading cause of blindness worldwide and has a significant impact on quality of life.^175^ About 80% of AMD patients are diagnosed with early dry subtypes, characterized by the formation of drusen under RPE, thickening of the Bruch membrane, and accumulation of lipofuscin in RPE.^176^ A study in 2010 showed that over 40% of the volume of drusen is composed of lipid‐containing particles.^177^ Similarly, Rodriguez et al.^178^, ^179^ found that 7‐ketocholesterol (7KCh), the main component of ox‐LDL, is mainly located in the outer layer of the retina. Therefore, we believe that the occurrence and development of AMD are closely related to cholesterol metabolism.

**FIGURE 2:**
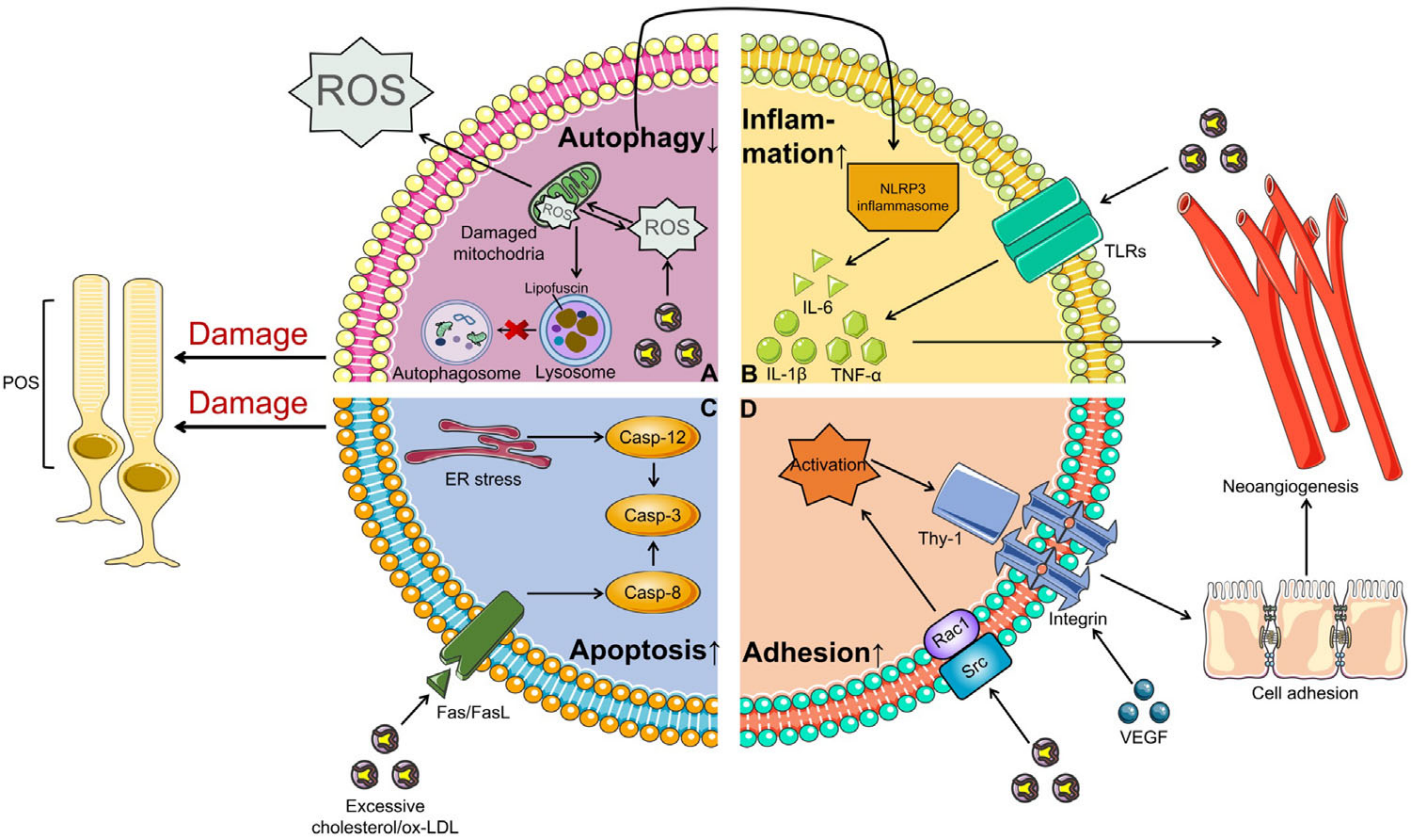
Effects of the accumulation of excess cholesterol/ox‐LDL in AMD. Excessive cholesterol is easily oxidized to ox‐LDL in the retina, which is the main component of 7KCh and widely affects multiple cellular processes. (A) First, under the condition of excessive lipid accumulation, oxidative stress can be triggered, leading to the mitochondrial damage in RPE cells, thereby increasing oxidative stress. Finally, some oxidation products polymerize, and lipofuscin accumulates in the lysosomes of cells, affecting the phagocytosis of lysosomes against autophagy and resulting in the impact on autophagy in the POS. In addition, decreased autophagy and mitochondrial damage can activate NLRP3 inflammasomes and further release inflammatory factors. (B) Second, excessive cholesterol activates NF‐κB pathway by binding to TLRs, increasing the expression of inflammatory factors, such as IL‐6, IL‐1β, and TNF‐α. (C) Third, the activation of Fas/FasL signals and ER under stress can increase the activities of Caspase‐3, ‐8, and ‐12 that promote apoptosis, leading to the increase of apoptosis of POS. (D) Fourth, the activation of Rac1 and Src by 7KCh leads to VEGF‐dependent expression of Thy‐1 and induces cell adhesion and migration, resulting to vascular rupture and angiogenesis with the increased expression of inflammatory factors. TLRs, Toll‐like receptors; VEGF, vascular endothelial growth factor; FasL, Fas receptor‐Fas ligand; POS, outer segment photoreceptor; NLRP3, NOD‐like receptor protein 3.

Currently, many studies have shown that 7KCh has a significant impact on the pathogenesis of AMD (Figure 2). First, concerning autophagy, P62, one of the targets of autophagic degradation, represents the autophagic flux of cells. Some studies have indicated that the level of P62 protein increases in 7KCh‐treated RPE cells, indicating that 7KCh can inhibit autophagy.^180^ Regarding the mechanism, we suspect that under conditions of high oxygen consumption and accumulation of lipid peroxidation products, oxidative stress is triggered, damaging mitochondria in RPE cells, in turn, increasing oxidative stress. Finally, some oxidation products polymerize, and lipofuscin accumulates in the lysosome of the cell, affecting the lysosomal phagocytosis of autophagy and leading to a decrease in the autophagic flux of RPE cells (Figure 2A).^181^, ^182^, ^183^ Second, in terms of inflammation, 7KCh can induce the expression of inflammatory cytokines, such as IL‐6, IL‐1β, and TNFα, mainly by activating the NF‐κB pathway.^44^, ^184^, ^185^ NF‐κB, as a transcription factor, is critical in mediating the expression of cytokines and other inflammatory markers. Huang et al.^185^ showed that inhibiting the NF‐κB pathway suppresses 7KCh‐induced inflammation and provides significant protection against 7KCh‐induced cell death. In addition, other signaling pathways, such as extracelluar signal‐regulated kinase (ERK), can also cause the increases of inflammation.^180^ Simultaneously, with a decrease in autophagy, the accumulation of ROS can induce the activation of NOD‐like receptor protein 3 (NLRP3) inflammatory bodies in human ARPE‐19 cells,^186^, ^187^ leading to a further enhancement of the cellular inflammatory response (Figure 2B).^188^ Third, in terms of apoptosis, 7KCh is also associated with apoptosis. Previous studies have shown that in ARPE‐19 and rat neuro‐retinal R28 cell cultures, 7KCh significantly increases the activities of caspase‐3, ‐8, and ‐12 that promote apoptosis.^189^, ^190^, ^191^ In addition, a study showed that the injection of 7KCh into the rat retina can induce apoptosis of photoreceptors and RPE cells, leading to the detachment of outer microvilli (Figure 2C).^180^ Therefore, we conclude that 7KCh can affect cell viability and normal physiological functions by regulating autophagy, inflammation, and apoptosis, ultimately accelerating AMD progression. In addition, there is some evidence that 7KCh can also affect cell adhesion, migration, and angiogenesis (Figure 2D), which requires further exploration.^192^

#### 7.1.2 Role of cholesterol metabolism regulation

After understanding the impact of excessive cholesterol on REC, potential treatment options have also emerged. Therapeutic goals can be achieved by reducing cholesterol biosynthesis and increasing cholesterol efflux. To achieve reduced cholesterol biosynthesis, it is necessary to inhibit the key rate‐limiting enzymes in the synthesis pathway. Although studies have shown that statins do not increase or reduce the risk of AMD,^193^, ^194^ a recent study has demonstrated that simvastatin can inhibit the biosynthesis of retinal cholesterol, which is valuable for future clinical research on statins as potential treatment methods for AMD.^195^ Additionally, DHCR24 inhibitors can also reduce the TC content in the retina.

However, the cholesterol efflux pathway is worth exploring due to its complex nature. In the retina, cholesterol efflux from cells is largely dependent on ABCA1/ABCG1. Before cholesterol is expelled from cells, it needs to be transported to the mitochondria and metabolized into steroids through the cytochrome P450 enzyme in the mitochondria. This process involves a complex of proteins, including the steroidogenic acute regulatory protein (StAR), 18‐kDa translocator protein (TSPO), voltage‐dependent anion channel (VDAC) and possibly the adenine nucleotide channel. STAR is divided into 15 different members and 6 subfamilies; STARD1 and STARD3 in the STARD1 subfamily are considered to play important roles in lipid transport and metabolism.^196^ STARD1 is a mitochondrial protein that regulates steroid production by transferring cholesterol from the outer mitochondrial membrane to the inner mitochondrial membrane and converting it into pregnenolone via the cytochrome P450 enzyme.^197^ Moreover, Borthwick and coworkers^198^ showed that STARD3 in ARPE‐19 cells can enhance cholesterol efflux, which is located on the membrane of the late endosome and transfers cholesterol from the late endosome to the ER and mitochondria.^199^ Almarhoun et al.^200^ found that the overexpression of STARD3 in ARPE‐19 cells can enhance cholesterol efflux in RPE cells by upregulating the expression of ABCA1 and reducing the accumulation of ox‐LDL, which ultimately inhibits oxidative stress and inflammation induced by ox‐LDL. Regarding TSPO, as a mitochondrial protein, it has the same effect as STARD1. TSPO combines with other proteins, such as STARD1 and VDAC, forming a multiprotein complex responsible for cholesterol transport.^201^ Some studies have shown that the activation of TSPO in mice leads to an increase in cholesterol efflux from RPE cells, a decrease in the levels of lipids (cholesterol, triglycerides, and phospholipids) in the retina, an increase in the expression of cholesterol‐related genes (LXR, ABCA1/ABCG1, and ApoE) and the inhibition of inflammation.^166^, ^202^, ^203^, ^204^ Simultaneously, TSPO can regulate the activity of STARD1,^205^ but the relationship between TSPO and STARD3 remains unknown. We previously reported that LXR plays a crucial role in cholesterol efflux by activating ABCA1 and ABCG1 to increase cholesterol efflux and inhibit inflammation. Through further investigation, we found that the activation of LXR can reduce the apoptosis of RPE cells induced by oxysterol by maintaining mitochondrial membrane stasis, which jointly activates mitochondrial autophagy via the mTOR/p62 pathway to promote mitochondrial metabolism.^206^ In addition to the above regulatory proteins, other molecular signals that increase the expression of ABCA1/ ABCG1 can also promote cholesterol efflux from cells and reduce the accumulation of ox‐LDL, delaying disease progression.

### 7.2 Corneal diseases

Like AMD, corneal diseases are also caused by the accumulation of cholesterol. Maintaining normal vision relies on corneal clarity. Excessive cholesterol accumulation in the corneal stroma can cause opacity, thus affecting clarity. In some cases, corneal transplantation is necessary to restore normal vision. In the cornea, collagen and proteoglycans form a dense connective tissue called the corneal stroma, which contains corneal cells. The outer and inner surfaces of the corneal stroma are covered by multiple layers of corneal epithelium and a single layer of corneal endothelial cells, respectively. Our findings indicate that ABCA1, ApoA1, ApoD, ApoE, and LCAT are present in the cornea, keratocytes, epithelial cells and endothelial cells.^171^, ^207^ Certain inherited diseases, including Tangier disease, familial LCAT deficiency, and familial ApoA1 deficiency, result in varying degrees of corneal opacity due to corneal cholesterol accumulation.^208^, ^209^, ^210^

In addition to mutations in RCT‐related genes, the mutation of UbiA prenyltransferase domain‐containing protein‐1 (UBIAD1) also leads to cholesterol accumulation.^211^ UBIAD1 is a member of the UbiA superfamily of integral membrane prenyltransferases. In animals, UBIAD1 can catalyze generation of menaquinone‐4 by transferring the 20‐carbon geranylgeranyl moiety from geranylgeranyl pyrophosphate (GGpp). Under normal conditions, to prevent the excessive accumulation of cholesterol, sterols can induce HMGCR to combine with ER‐anchored INSIG‐1 and INSIG‐2, then some ubiquitin ligases ubiquitin HMGCR together with other cofactors, leading to HMGCR degradation in the proteasome, which is called the ER‐associated degradation (ERAD) of reductase.^212^, ^213^, ^214^, ^215^, ^216^, ^217^ However, sterol also induces the binding of reductase to UBIAD1, inhibiting the ubiquitination step of ERAD reductase and allowing continued mevalonate production.^218^ Concurrently, the accumulation of GGpp in the ER membrane induces the release of UBIAD1 from the reductase, alleviating the inhibition of ERAD and facilitating the transport of UBIAD1 from the ER to the Golgi.^219^ It is precisely due to the above regulatory mechanisms that cholesterol in the corneal stroma can always maintain a stable level for a long time. However, when *Ubiad1* is mutated, the transfer of UBIAD1 mediated by GGpp is inhibited, leading to the inhibition of ERAD and the accumulation of cholesterol, eventually resulting in corneal opacity, also known as Schnyder corneal dystrophy (SCD) (Figure 1).^219^ Currently, we are seeking drugs that accelerate the degradation of UBIAD1 related to SCD, trigger the transport of variants from ER to Golgi apparatus or prevent their interaction with reductase, which may prevent cholesterol accumulation and corneal opacity related to SCD.

### 7.3 Cataract

#### 7.3.1 The relationship of cataract and cholesterol metabolism

Unlike AMD and corneal diseases, cataracts seem to rely on high concentrations of cholesterol.^220^ To achieve clear vision, we depend on the lens to refract light onto the retina. Therefore, lens transparency is crucial for clear vision. A recent study has found that the long‐term inhibition of cholesterol biosynthesis in the lens reduces the rate of fiber proliferation and differentiation, increasing the risk of lens opacity.^168^ This suggests that cholesterol is beneficial for the lens.

The lens is different from the retina, it is an avascular structure, which means not all nutrients in the blood can easily penetrate it. Dietary cholesterol does not affect the cholesterol content in the lens.^221^ In addition, the cholesterol content of the aqueous humor is extremely low, which is negligible compared with that of plasma. Therefore, De Vries et al.^222^ concluded that cholesterol accumulation in lens depends on in situ synthesis. This perspective implies that cholesterol is valuable for the lens. So why is cholesterol beneficial for the lens?

Cholesterol plays a crucial in maintaining lens transparency. First, cholesterol is necessary for the formation of cytoplasmic membranes during lens epithelial cell (LEC) differentiation.^223^ Cholesterol in the cell membrane can form a highly hydrophobic barrier between cells to reduce light scattering and maintain lens transparency by maintaining an appropriate volume and water content in lens fibers.^224^ Second, the high cholesterol content in the membranes maintains a low oxygen partial pressure. When the cholesterol content of the cells is high, the oxygen permeability coefficient of the membrane is lower than that of the water layer of the same thickness, which is crucial for lens transparency.^225^, ^226^ Thus, a high cholesterol level is beneficial for the lens. Once cholesterol biosynthesis is affected, lens transparency cannot be guaranteed, and the risk of cataracts will increase. For example, approximately 20% of patients with Smith Lemli Opitz syndrome who are deficient in DHCR7 have cataract^227^; the missense mutation of lanosterol synthase (LSS, cyclinase in cholesterol biosynthesis) causes cataract in pedigree analysis of close relatives of Caucasian descent; cataracts occur in approximately 30% of patients with mevalonate aciduria caused by mevalonate kinase deficiency.^228^ Therefore, maintaining a high cholesterol content is crucial for the normal function of the lens.

#### 7.3.2 Role of cholesterol metabolism regulation

Given the crucial role of cholesterol in the lens, the metabolic regulation of cholesterol within the lens has become a focus of research. At present, studies have found that LSS, as a key rate‐limiting enzyme in cholesterol synthesis, plays a crucial role in cataractogenesis.^229^ The rs2968 polymorphism of *Lss* gene is associated with karyotype risk of age‐related cataracts in the Chinese population.^230^ However, as a causal gene of cataracts, the role of *Lss* in the lens remains largely unknown. Now, we summarize the role of LSS based on existing research findings. First, LSS, as a key rate‐limiting enzyme in the cholesterol synthesis pathway, plays a critical role in maintaining high cholesterol content in the lens.^170^ Second, LSS plays an important role in lens development. Zhao et al.^169^ found that blockage of primary fiber differentiation and delay of secondary fiber differentiation, as well as incomplete karyolysis, occur in mice with a homozygous *Lss*G589S/G589S mutation. Simultaneously, the polarity of the lens fiber cells from top to bottom is lost, potentially leading to a disorder in lens fiber differentiation.^169^ In addition, LSS can promote the synthesis of lanosterol in the biosynthetic pathway, which decomposes large protein aggregates into small protein aggregates to reverse protein aggregation in cataracts.^231^ Third, LSS also have an effect on oxidative stress. Oxidative stress regulates various cellular processes related to cell survival, such as proliferation, differentiation, aging, and death,^170^ which is the major cause of age‐related ophthalmopathy.^232^, ^233^ In a recent study, researchers increased oxidative stress in cells by exposing LECs to UV‐B and found that LSS reduces UV‐B‐induced apoptosis and exerts an antioxidant effects in the early stages of cell damage.^229^ In another study, Ishida et al.^170^ analyzed that *Lss* deficiency may lead to the reduction of antioxidant gene expression. Therefore, LSS is highly anticipated due to its important functions. However, due to its complex physiological properties, it has not yet been applied to clinical practice, and further exploration is needed in the future.

### 7.4 Dry eye syndrome

DES is a disorder of tear secretion that causes eye discomfort, eye fatigue and visual impairment due to insufficient or excessive evaporation of tears.^234^ The tear film is composed of lipid, aqueous, and mucin layers, among which the lipid layer protects the eye from environmental pressure.^235^ The meibomian gland, the main component of the tear film lipid layer, is responsible for the secretion of lipids, including cholesterol and CEs. Dysfunction of the Meibomian gland is a common ocular ailment that results in DES by causing local dyslipidemia. Hence, systemic dyslipidemia may be associated with the pathogenesis of DES.^236^ A recent study investigated the relationship between dyslipidemia and DES in a middle‐aged Korean population. They defined dyslipidemia as TC > 240 mg/dL, HDL‐C < 40 mg/dL, LDL‐C > 160 mg/dL, or triglycerides > 200 mg/dL. Moreover, they discovered that the odds ratio for DES in men with dyslipidemia was 1.29 (95% confidence interval, 0.97−1.71) compared with men without DES. However, no significant correlation between female dyslipidemia and DES was observed, suggesting a potential association between dyslipidemia and DES prevalence in Korean men, but not women.^236^ In another study, Aldaas et al.^172^ examined the relationship between statin use and DES. They found that the local use of statins is beneficial for the treatment of dry eyes related to blepharitis, but the risks of DES for low‐intensity, medium‐intensity and high‐intensity statins in the treatment of blepharitis are similar, which suggests that the effect of statins may not be dose‐dependent, or the correlation between statins and DES may be false.^172^ Therefore, additional research is needed to clarify the association between dyslipidemia and DES.

### 7.5 Diabetic retinopathy

DR is a serious complication of diabetes, characterized by retinal ischemia and neurodegeneration, eventually leading to vision loss.^237^ According to the development of DR, it is be classified into nonproliferative diabetes retinopathy (NPDR) and proliferative diabetes retinopathy (PDR). NPDR is characterized by changes in the retinal vascular system, such as increased permeability. Untreated, these changes induce ischemia, leading to neovascularization, commonly referred to as PDR. Opreanu et al.^238^ previously demonstrated that abnormal retinal lipid metabolism can induce retinal degeneration by activating chronic inflammation. Recent studies indicate that dyslipidemia and lipid peroxidation are significant contributors to DR pathogenesis.^239^, ^240^ These findings suggest that regulating related metabolism could delay or prevent vision loss attributed to DR. Consequently, enhancing RCT is advantageous for preventing and treating DR. LXR, a crucial receptor in the RCT pathway, its decreased signal transduction is a hallmark feature of type 2 diabetes, which can lead to retinal cholesterol metabolism disorder and increased production of proinflammatory cytokines.^241^ A study demonstrated that LXR is involved not only in regulating lipid metabolism but also in the insulin signaling pathway, influencing glucose metabolism.^242^ Given the effectiveness of LXR, it may emerge as a potential target for future DR treatment.

While our current grasp of cholesterol metabolism in the eye is not exhaustive, we believe eye diseases are closely linked to abnormal cholesterol metabolism. Simultaneously, we have identified numerous potential therapeutic targets and summarized them in this review (Table 4),^243^, ^244^, ^245^ with the aim of fostering a more comprehensive understanding of disease prevention and treatment in the future.

**TABLE 4:** The functions and effects of potential therapeutic targets for eye diseases.

Disease	Signals	Functions	Roles	References
AMD	Statins	HMGCR↓ IL‐6/8↓	Cholesterol synthesis↓ Inflammation↓	^243^
	The inhibitors of miRNA	ABCA1↑	Cholesterol efflux↑	^244^
	AMPK	ABCA1/ABCG1↑	Cholesterol efflux↑	^245^
	Oxysterols/LXR agonists	ABCA1/ABCG1↑ The mTOR/p62 pathway↑ The NF‐κB pathway↓	Cholesterol efflux↑ Autophagy↑ Inflammation↓	^44^, ^206^
	Gypenosides	ABCA1/ABCG1↑ CYP46A1↑ The NF‐κB pathway↓ The ox‐LDL accumulation↓	Cholesterol efflux↑ Inflammation↓ Antioxidant capacity↑	^184^
	STARD3	ABCA1↑ IL‐1β↓ TNF‐α↓ The ox‐LDL accumulation↓	Cholesterol efflux↑ Inflammation↓ Antioxidant capacity↑	^200^
	TSPO	ABCA1/ABCG1↑ CYP46A1↑ IL‐1β↓ TNF‐α↓ The ox‐LDL accumulation↓ Lipogenesis↓	Cholesterol efflux↑ Inflammation↓ Antioxidant capacity↑ Cholesterol synthesis↓	^166^, ^202^, ^204^–
Cataract	LSS	Lipogenesis↑ Lenosterol↑	Cholesterol synthesis↑ Protein aggregation↓	^169^, ^170^
DR	LXR agonists	ABCA1/ABCG1↑ GLUT4↑	Cholesterol efflux↑ Blood glucose↓	^242^

## 8 CONCLUSIONS AND PROSPECTS

Widely distributed throughout the human body, cholesterol plays a crucial role in various physiological processes. For example, in the brain, cholesterol synthesized by oligodendrocytes and Schwann cells mainly exists in the form of myelin sheath, a vital component for the nervous system's normal functioning.^246^ A well‐regulated cholesterol metabolism is also crucial for maintaining the body's normal functioning and keeping cholesterol levels stable. Once cholesterol synthesis or efflux is impaired, it may lead to the occurrence of diseases. Our studies reveal that defects in cholesterol metabolism are closely linked to various diseases, including CVD, tumors, neurological disorders, immune system issues, and eye diseases. Excessive accumulation of cholesterol often promotes the progression of diseases and even plays a role as a “marker.” Cholesterol overload can induce elevated oxidative stress, heightened inflammatory responses, reduced autophagy, and increased apoptosis in cells through various signaling pathways, ultimately accelerating the development of diseases. However, high levels of cholesterol within the lens are beneficial for maintaining lens transparency, and inhibiting cholesterol synthesis often leads to lens opacity, resulting in cataracts. It is important to note that in elderly individuals, surpassing the cholesterol solubility threshold can result in the formation of cholesterol crystals, causing pathological manifestations.^247^

Although the exact mechanism by which cholesterol metabolism plays a role in the pathogenesis of diseases is not yet clear, reducing cholesterol accumulation undoubtedly contributes to the treatment of diseases. On the one hand, we can employ inhibitors to decrease cholesterol production by targeting key enzymes in the cholesterol synthesis pathway, like statins, widely utilized in clinical practice for their antioxidant and anti‐inflammatory properties. Nevertheless, further efforts are required to minimize the side effects of statins and discover more effective and safe medications for modifying cholesterol metabolism. On the other hand, mounting evidence indicates the significance of the RCT pathway in cholesterol metabolism. Activating this pathway to enhance cholesterol efflux offers a potential avenue for clinical practice. Specific receptors in the RCT pathway, like LXRs, have emerged as potential therapeutic targets. Currently, the development of selective LXR receptor agonists that do not affect liver and plasma triglyceride levels has become a research focus. In the near future, LXR agonists could evolve into tailored therapeutic targets for addressing cholesterol metabolism disorders.

However, some unresolved issues persist concerning cholesterol metabolism. Due to barriers, cholesterol in the brain and retina primarily results from in situ synthesis. It remains uncertain if cholesterol in these regions is regulated by the entire body, posing a challenge in explaining the relationship between plasma cholesterol levels and disease risk. Additionally, the influence of cholesterol metabolites on cholesterol homeostasis merits further investigation, including the role of 24‐OHC and CYP46A1 in diseases and their effect on cholesterol turnover rate.

## AUTHOR CONTRIBUTIONS

J. G. collected literature and drafted the manuscript. S. C., Y. Z., J. L., and L. J. drew the figures and tables. L. H. and K. Y. reviewed and made significant revisions to the manuscript. Y. Y. and X. C. finalized the manuscript and contributed to funding acquisition, validation, and resources. All authors have read and approved the final manuscript.

## CONFLICT OF INTEREST STATEMENT

The authors declare no conflict of interest.

## ETHICS STATEMENT

Not applicable.

## Data Availability

Not applicable.
